# Evidence of Spinal Cord Damage in Human T Cell Lymphotropic Virus Type 1-Infected Subjects without Motor Disability

**DOI:** 10.4269/ajtmh.25-0126cor

**Published:** 2026-04-02

In the original research paper, “Evidence of Spinal Cord Damage in Human T Cell Lymphotropic Virus Type 1-Infected Subjects without Motor Disability” by José Abraão Carneiro Neto, et al., 2026, (https://doi.org/10.4269/ajtmh.25-0126), an error was introduced prior to publication with one of the funding agencies supporting this work (Fundação Maria Emília) being inadvertently omitted from the Financial Support section. The authors sincerely regret this error. The paper has since been corrected. The corrected statement is provided below:

1. “Financial support: This work was supported by the Fundação Maria Emília and Fundação de Amparo à Pesquisa do Estado da Bahia under grant number INC0003/2019.” (page 332)

